# The Effect of a Single Session of Functional Electrical Muscle Stimulation During Walking in Patients with Hemiparesis After Stroke: A Pilot Pre–Post Study

**DOI:** 10.3390/jfmk10040480

**Published:** 2025-12-16

**Authors:** Dmitry Skvortsov, Danila Lobunko, Natalia Grebenkina, Galina Ivanova

**Affiliations:** 1 Federal State Budgetary Institution “Federal Center of Brain Research and Neurotechnologies” of the Federal Medical Biological Agency, Moscow 117321, Russia; 2 Research and Clinical Centre of Moscow, Moscow 107031, Russia

**Keywords:** electric stimulation therapy, stroke rehabilitation, gait, biomechanical phenomena, electromyography, hemiplegia, walking speed

## Abstract

**Background:** Functional electrical stimulation (FES) is widely used in post-stroke rehabilitation to restore motor activity and improve walking. However, the immediate effects of a single FES session on gait biomechanics and muscle activity remain insufficiently studied. This pilot study aimed to evaluate the direct neuromotor effects of a single multichannel FES session during walking in patients with post-stroke hemiparesis. **Methods:** Eight patients with hemiparesis in the early or late recovery period after ischemic stroke underwent gait biomechanics and electromyography (EMG) assessment before and immediately after a single 30 min FES session. FES was applied to the tibialis anterior, gastrocnemius, quadriceps femoris, and hamstring muscles of the paretic limb during walking, synchronized with gait phases. Spatial-temporal, kinematic, and EMG parameters were recorded using an inertial system. Pre- and post-intervention data were compared using paired tests (a paired *t*-test or the Wilcoxon signed rank test, *p* < 0.05), while the standardized effect sizes (Cohen’s d) were calculated for all pre-post comparisons. **Results:** A significant decrease was observed in the single support phase of the paretic limb after FES (*p* < 0.05). Knee joint movement amplitude increased significantly in the nonparetic limb. Surface EMG amplitudes decreased in the tibialis anterior of the nonparetic limb and in the hamstring and gastrocnemius of the paretic limb (*p* < 0.05). No significant changes were detected in overall gait speed, rhythm, or phases of muscle activity peaks. **Conclusions:** A single session of multichannel FES induces neuromotor changes reflected by redistribution of muscle activity and compensatory adjustments in gait biomechanics without immediate improvement in global kinematic parameters. The direct biomechanical changes in the gait function can be interpreted as evidence of the onset of fatigue. The procedure demonstrated good tolerability and safety, confirming its feasibility for early post-stroke rehabilitation.

## 1. Introduction

Stroke holds a leading position in the mortality structure and among the main causes of disability [1]. The growing stroke incidence, the clinical features of the disease and the severity of its sequelae place utmost importance on optimization of its treatment and subsequent rehabilitation [2,3]. Walking recovery is one of the principal goals of post-stroke rehabilitation [4]. Studies show that acquired post-stroke asymmetric walking patterns persist in stroke survivors even in the chronic phase of the disease [5,6,7]. Regaining normal walking—an important factor for safe and effective social functioning—is a complex problem even with intensive rehabilitation measures [8].

According to the current guidelines, one of the essential rehabilitation interventions for stroke survivors is functional electrical muscle stimulation (FES) [9].

The use of FES is based on restoring motor activity by activating the nervous system [10], generating necessary changes not only at the level of the muscular apparatus, but also in the central nervous system [11]. In fact, application of low electric current modulates the conduction velocity, axon growth, and peripheral nerve myelination [12], while the central effects of FES induce peripheral efferent activation, thus improving the contractile strength, resistance to muscle fatigue [13], movement coordination [14], and muscle mass [15].

Despite the positive results achieved with FES, there are still questions regarding FES treatment methodology and effectiveness evaluation. For instance, the optimal duration of a FES session has not been established: according to some authors, it should last 15 to 30 min [16], while others recommend up to 30–45 min [17]. There are also disagreements on the overall duration of a FES treatment course: from 3 weeks in some protocols [18] to over 12 weeks in others [19].

The same applies to evaluation of treatment effectiveness. Traditionally, it is a common practice to evaluate changes in walking speed and in the strength of stimulated muscles [20,21]. These parameters, however, cannot reflect all changes in gait biomechanics, which, in their turn, may be necessary for potential treatment adjustment.

In addition, there are a number of obstacles that prevent medical professionals from using FES: lack of special training, expertise and resources, perception of the method as unsuitable for some stroke patients, as well as personal preferences of the specialist [9].

No doubt, for both medical professionals and the patients it is important to understand and roughly assess the procedure effectiveness after the very first session. A single administration of FES in stroke patients was studied primarily in terms of neuromuscular and corticomotor effects. A study in patients who suffered a stroke more than 6 months previously [22] showed that a single FES session caused a positive motor response: it increased the plantar flexor corticomotor symmetry, which correlated with improved ankle moment symmetry between paretic and nonparetic limbs, although the authors were unable to establish a causal relationship between the observed effects. Another study [23] evaluated direct changes in the electrical resistance of muscles after a single FES session in stroke survivors. The identified changes were shown to be possibly related to the developing muscle hypertrophy.

In a comparative effectiveness study of FES versus a standard set of physical exercises [24], the FES group demonstrated improved balance and cognitive functions, probably due to regulation of oxidative stress markers.

Assessments of gait biomechanics before and after a course of FES [25] for thigh and lower leg muscles on the paretic side in patients with subacute stroke showed almost no objective changes in the functional status. FES sessions were conducted over a two-week period (10–11 FES sessions, with a mean duration of 25.5 min).

It remains unclear what effects a single FES session can have on the muscle function and how this function changes under FES. Assessment of paretic leg muscle response to FES in a walking patient can probably help predict the effectiveness of the entire FES treatment course. Such evaluation of treatment effectiveness is of significant economic importance.

The objective of this study was to evaluate the direct effects of a single FES session on the function of stimulated muscles in patients with post-stroke hemiparesis. We considered the following three hypotheses for the direct effect of FES: an increase in muscle activity; a change (normalization) in the phase of the main peak of activity in the gait cycle; a decrease in muscle activity without any change in the phase.

## 2. Materials and Methods

### 2.1. Study Design

This study used a single-group pre-post design, in which each participant underwent a biomechanical gait assessment immediately before and after a single multichannel FES session. This design allowed the assessment of acute neuromotor responses to FES without a long-term follow-up or a control group. This approach is aligned with the research objectives of the pilot study.

### 2.2. Study Patients

The study included 8 patients with hemiparesis after a cerebral stroke, in the early or late recovery period. The final sample included 1 female and 7 male patients: 4 with right-sided hemiparesis and 4 with left-sided hemiparesis. The mean age of the patients was 52.8 ± 10.7 years, and the mean time from stroke to study assessments was 130.6 ± 100.9 days. The mean proximal lower limb muscle strength on the paretic side was graded 3.75 ± 0.46, whereas the distal muscle strength was 2.75 ± 1.03 on the MRC scale. The mean lower limb muscle tone was 0.5 ± 0.53 on the modified Ashworth scale. The mean Dynamic Gait Index score was 14.5 ± 2.07, and the mean Timed Up and Go test result was 32.9 ± 19.6 s.

Each patient underwent an initial assessment of gait biomechanics, which included kinematic parameters and muscle electrical activity (EMG). The patients were then administered a single session of multichannel FES to stimulate the following muscles in the paretic limb: tibialis anterior (TA), gastrocnemius (GM), quadriceps femoris (QF), and hamstring (HM). The stimulation lasted 30 min if well tolerated by the patient. No adverse events were recorded. None of the patients complained of pain during the session. The patients reported mild fatigue after the session. The second biomechanical gait assessment was performed immediately after the FES session and according to the same protocol as the initial one.

### 2.3. Eligibility Criteria for Patients

Inclusion criteria: patients with hemiparesis in the early or late recovery period after a first-time hemispheric ischemic stroke; age up to 75 years; functionally ready for verticalization; adequate response to orthostasis test; no risk of falling. Rankin score of at least 3 points; able to understand and follow simple instructions; stable vegetative and hemodynamic parameters; decreased muscle strength in the lower limb: up to 3 points on the MRC; with manifestations of abnormal muscle tone up to 1.1+ points on the modified Ashworth scale. Absence of deep sensitivity disorders in the lower limbs; absence of a decompensated somatic disorder, no evidence of ischemic changes on electrocardiogram or heart failure (Killip class II or lower); apart from stroke, no other diseases of the central or peripheral nervous system accompanied by neurological deficits (sequelae of injuries, tumors, polyneuropathy, etc.); no EEG evidence of epileptic activity; absence of any orthopedic pathology (joint deformities or contractures, severe pain syndrome, amputated limbs on the paretic side, no operations on paretic upper limb joints with the use of medical hardware, the ability to walk independently at a free pace for 15 min or more etc.).

Exclusion criteria: Inadequate cardiovascular response during the assessment or training; individual intolerance to transcutaneous electrical stimulation; patient’s refusal to continue therapy; deterioration of neurological and/or somatic condition during the study; presence of an implanted pacemaker, artificial pacemaker or other medical device incompatible with the procedure near the stimulation zone; severe pain in the paretic lower limb at rest or during movement, preventing exercise; severe cognitive impairment, psychoemotional agitation, hysterical reactions or pseudobulbar affect; severe speech disorder that prevents understanding and following instructions; skin lesions or skin diseases in the area of proposed stimulation in the absence of a dermatologist’s opinion; venous thrombosis of the lower limbs without signs of recanalization or arterial thrombosis; established diagnosis of epilepsy; Failure to comply with the study protocol or visit schedule, as well as early patient withdrawal from the study, were considered. Participants were recruited using convenience-based sampling and included stroke patients who met the inclusion criteria and provided a prior written consent to participate in the study. Because the study was pilot and exploratory in nature, a formal sample size calculation was not performed. A sample size of eight participants was considered sufficient to assess the feasibility of the protocol and to preliminarily estimate the direction and the size of the intervention effect in a one-group pre-post design.

All participants in this study had signed a written informed consent approved by the local ethics committee (11/25-04-22 dated 25 April 2022).

### 2.4. Methods of Biomechanical Assessment

Gait biomechanics was assessed using a Steadys system (Neurosoft, Ivanovo, Russian Federation). Seven inertial measurement units (IMUs) were used, each having two channels for EMG recording. The IMUs were attached to the sacrum and, on both legs, to the lateral middle third of the thigh, lateral lower leg above the ankle, and on the bridge of each foot. Before recording, calibration was performed with the patient standing upright. If necessary, an assistant helped the subject maintain the desired position for 2–3 s. Gait biomechanics was assessed under standardized laboratory conditions, while the patient was walking at a comfortable pace on a flat surface wearing their own shoes. All patients wore their own comfortable shoes. Walking was performed along the long side of the laboratory (15 m) at room temperature, with turns at the end of the walkway and then continued walking. This was repeated several times. The walking speed was not specified, and the patients chose their own comfortable speed. During the biomechanical study and FES, a laboratory employee was always present next to the patient.

The IMUs sent the captured data to a computer via a Wi-Fi network. Special software recorded the parameters of interest while the patient was independently walking. Automatic data analysis was done using a neural network [25]. Data capture was completed after reaching 30 gait cycles. The software identified the gait cycles of paretic and nonparetic limbs and calculated other gait cycle parameters. Spatial, temporal, kinematic, and biomechanical parameters were recorded.

Gait biomechanics was reassessed immediately after the FES session. No special rest period was allowed. However, all patients had a short break in a standing position for 4–4.5 min after the end of FES and before the start of the reassessment. The break was necessary to remove the stimulating electrodes and units and install the IMU sensors. None of the patients in this study used a cane or other walking aids, or orthoses.

Electromyography (EMG) was recorded from the main flexor and extensor muscles: tibialis anterior (TA), both gastrocnemius (GM) muscles, quadriceps femoris (QF), hamstring (HM). EMG was recorded using disposable surface Mederen electrodes (Israel). The EMG channels collected native data at a frequency of 2000 Hz and sent them to the computer via WiFi. Subsequent processing included a high-pass filter with a cutoff frequency of 5 Hz. Smoothing of the EMG profile after its rectification was performed with a constant component of 200 ms. Notch filter (50 Hz) was not used since the sensor operated independently of the battery.

We used a modified technique for muscle electrode placement, different from the SENIAM standard. The FES technique we used stimulates large muscle groups, so our goal was to obtain EMG data not from individual muscles, but rather from a group, in order to capture muscle activity across the largest possible motion range of flexor and extensor muscles. Therefore, we applied electrodes transversely across the thigh in the middle third, both anteriorly (the quadriceps femoris and the hamstring). For the gastrocnemius muscle, one electrode was placed at the midpoint of the lateral head, and the second at the midpoint of the medial head. Only the tibialis anterior muscle was recorded separately.

Such placement of EMG electrodes also allowed them to remain on the patient during FES. This was crucial in our study, as changing the position of any of the EMG electrodes would have made comparisons of the absolute amplitude of muscle activity inaccurate.

The assessed temporal parameters of the gait cycle (% of gait cycle) included stance phase, single support, and the beginning of terminal double limb stance phase (beginning of the gait cycle of the other limb).

The kinematic parameters were recorded for the hip, knee, and ankle joints in the sagittal plane (flexion-extension) to further generate goniograms and calculate maximum joint movement amplitudes per gait cycle. Muscle EMG was analyzed for maximum activity (in µV) developed in the gait cycle.

All measurements were performed at the same time of day to exclude the influence of circadian rhythms.

### 2.5. FES Procedure

Functional electrical stimulation was conducted according to a standardized protocol using the stimulators of the Steadys system (Neurosoft, Ivanovo, Russian Federation). For the FES training sessions, we used modified IMUs where two EMG channels were replaced with two FES channels.

The stimulation devices had the same housing as those used for diagnostics. Therefore, on the paretic leg, the devices on the elastic cuffs of the thigh and calf were replaced with the stimulating ones. Both foot sensors were removed. The sensors on the healthy side were used to determine the gait cycle and record movements in the hip and knee joints (EMG was not recorded). The stimulation devices on the paretic side were used both for muscle stimulation and to record the temporal characteristics of the gait cycle, as well as movements in the hip and knee joints. Movement kinematic data was used to monitor the stimulation process, while gait cycle data was used directly to precisely synchronize the stimulation channels with the gait process.

Stimulation was performed using adhesive electrodes (Fiab, Italy). The electrodes were positioned to cover a maximum possible area of the target muscle belly and secured above and below the middle of the belly. The dimensions of FES electrodes were 46 × 98 mm for the GM, QF, HM muscles, and 50 × 50 mm for TA.

**Figure 1:**
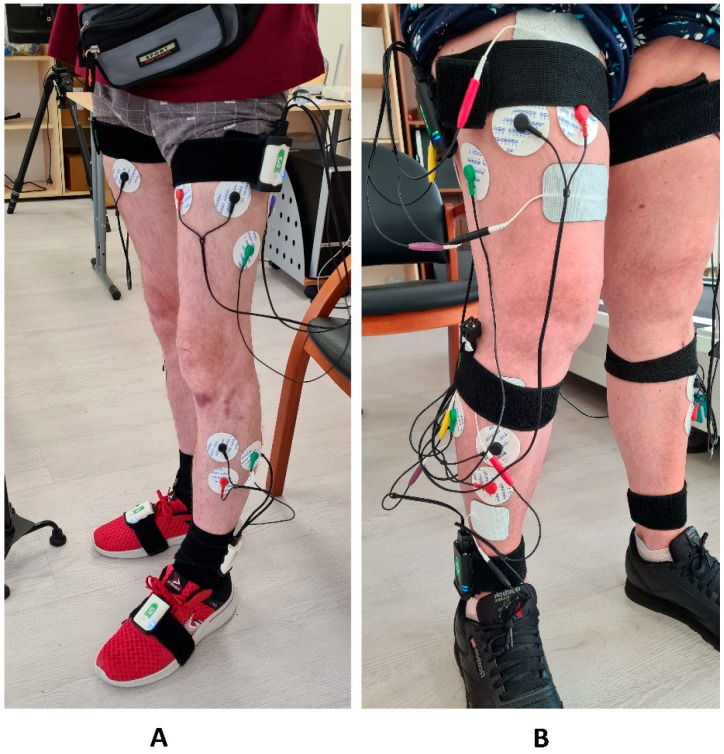
(**A**) On the left, the “Stedis” system is mounted on the patient for recording biomechanical gait parameters. Elastic cuffs, inertial sensors with EMG channels, and EMG electrodes with connecting cables are visible. (**B**) In on the right—setup of the stimulation system without removing the EMG electrodes and elastic cuffs placed on the sacrum, thighs, and shanks. Above and below the EMG electrodes are stimulation electrodes connected by cables to stimulation devices secured with elastic cuffs. The EMG electrode cables are coiled and fixed to the elastic cuffs.

The EMG electrodes were placed onto the most massive, middle part of the muscle belly (Figure 1). Such placement of electrodes was to ensure similar conditions for biomechanical assessments before and after the FES session: the EMG electrodes and the cuffs with sockets for IMUs did not change their position.

Each target muscle group was electrically stimulated with bipolar electric pulses at a frequency of 50 Hz and a pulse width of 200 µs [26]. The start and end of stimulation within the gait cycle were set in accordance with maximum activity of the muscle in a normal gait cycle [27]. As is customary for this method, the stimulation intensity (current) was set so as to meet two conditions: to produce a visible muscle response to the test stimulus and a corresponding movement in the joint (knee or ankle, depending on the target muscle). The current was gradually increased in trial stimulations until being set below the pain threshold for the muscle. This setting-up process was performed for each muscle. Synchronization of the stimulation beginning and end within the gait cycle was achieved using an automatic gait cycle detection algorithm [28]. For this purpose, data from the IMU on the lower leg (right above the lateral ankle) was automatically processed by a neural network to identify the gait cycle and assess other parameters.

The stimulation was performed while the patient was walking back and forth on a 15 m-long flat surface. The stimulation automatically stopped when the patient was turning (at the end of the walkway) and resumed when the gait cycle became steady again (usually at the second or third cycle after the turn). The stimulation continued for 30 min throughout the entire procedure. The average number of gait cycles performed by patients during a FES session was 836. The average mean current intensity was 74 mV for the tibialis anterior, 75 mV for the gastrocnemius, 69 mV for the quadriceps femoris, and 70 mV for the hamstring.

After the end of the FES session, the stimulators were replaced with IMUs for capturing biomechanics and the assessment of biomechanical parameters was immediately repeated (Figure 1).

### 2.6. Statistical Analysis

Statistical analysis was performed using Statistica 12 (StatSoft Inc., Tulsa, OK, USA). Normality of data distribution was assessed using the Shapiro–Wilk test. For normally distributed parameters, the pre-post differences were analyzed using a paired *t*-test. For nonparametrically distributed data, the Wilcoxon signed-rank test for related samples was used.

To quantify the magnitude of changes, regardless of their statistical significance, the standardized effect size (Cohen’s d) was calculated for all pre-post comparisons. The effect sizes were interpreted as minor (<0.20), small (0.20–0.49), medium (0.50–0.79) and large (≥0.80). The differences were considered significant at *p* < 0.05.

## 3. Results

### 3.1. Spatiotemporal Gait Parameters

The comparison of spatiotemporal gait parameters before and after intervention revealed a limited number of acute changes after the FES session. The only significant difference was a decrease in the single-stance phase of the paretic limb (*p* = 0.0156), indicating a transient decrease in stance stability or a protective unloading strategy on the affected side immediately after the stimulation.

The decrease in the single support phase of the paretic limb corresponded to a Cohen’s d of −1.16, indicating a large effect. The double support phase and the foot clearance of the paretic limb also demonstrated large effects (Cohen’s d of 0.84 and −1.21, respectively), whereas all other spatiotemporal parameters showed only small or minor effects.

In contrast, no significant changes were observed in other spatiotemporal characteristics. Step cycle duration, stance-to-swing phase ratio, double-stance duration, gait rhythmicity index, gait speed, foot rise height, and circumduction remained stable for both limbs. Thus, a single FES session did not alter the global temporal organization of gait in this assessment (Table 1).

### 3.2. Joint Kinematics

Analysis of the joint motion ranges revealed a significant increase in the flexion-extension amplitude in the knee joint of the non-paretic limb after the FES session (*p* = 0.0234).

The increase in the non-paretic knee movement amplitudes corresponded to a large effect size (Cohen’s d = 1.09), whereas the changes in the knee joint of the paretic limb were of a medium size (d = 0.71), and just small effects were observed in the hip and ankle joints of both limbs (d < 0.37). This change may reflect compensatory adjustments on the non-paretic side during walking after stimulation. Changes in the paretic limb did not reach statistical significance.

The movement amplitudes in the hip and ankle joints of both limbs remained unchanged. Overall, the kinematic pattern was characterized by a localized increase in mobility in the knee joint of the non-paretic limb, without significant changes in other joints. The movement amplitudes in the joints before and after the study intervention are presented in the upper part of Table 2.

### 3.3. Surface EMG Amplitude

Post-intervention analysis revealed a significant decrease in the surface EMG amplitudes for several muscles. In the paretic limb, a statistically significant decrease in the EMG amplitude was noted in the hamstring and gastrocnemius muscles (*p* = 0.0391 and *p* = 0.0391, respectively). In the non-paretic limb, a significant decrease in the EMG amplitude was observed in the tibialis anterior (*p* = 0.0078). These results are consistent with the development of acute neuromuscular fatigue after a session of multichannel electrical stimulation.

The observed decrease in the EMG amplitude was accompanied by large effect sizes for the anterior tibial muscle on the nonparetic side (Cohen’s d = −1.95) and the gastrocnemius muscle on the paretic side (d = −1.03), as well as medium effect sizes for the muscles of the posterior femoral group on both limbs (d ≈ −0.70 to −0.76). For other muscles, only small or minor effects were noted.

No significant changes in activation amplitude were observed for the remaining muscles. Despite the localized decrease in activity in several muscles, the overall pattern of muscle recruitment within the gait cycle remained recognizable. The corresponding EMG amplitude values are presented at the bottom of Table 2.

### 3.4. Phases of Maximum Muscle Activity

Analysis of the EMG peak phases revealed no statistically significant changes in muscle activation peaks for any of the studied muscles in either limb. For most muscles, the peak activation position within the gait cycle remained within the same phase intervals as before the intervention.

Overall, the temporal structure of muscle activation within the gait cycle was resilient to acute FES, indicating that a single session does not alter the underlying temporal pattern of neuromuscular gait control. The corresponding phase values of peak electrical activity before and after the intervention are presented in Table 3.

Maximum EMG amplitudes within the gait cycle (µV) and the phases of the activity maxima (% of gait cycle) were determined from EMG envelopes.

## 4. Discussion

This pilot study evaluated the effects of a single session of multichannel functional electrical stimulation of lower limb flexors and extensors on the gait and EMG parameters in patients with post-stroke hemiparesis. The key observations include (1) a statistically significant decrease in the single support phase of the paretic limb immediately after the FES session; (2) an increase in the flexion/extension amplitude in the knee joint of the nonparetic limb; (3) a decrease in the EMG amplitude of tibialis anterior on the healthy side, as well as of hamstring and gastrocnemius on the paretic side. Despite the pilot nature of the study and its small sample size, the observed significant post-intervention changes corresponded to medium or large effect sizes for key kinematic and EMG parameters, which confirms the physiological significance of the obtained results, even with the limited statistical power of the study.

The decrease in the single support phase on the paretic side immediately after the intervention contradicts the previously described stabilizing effect of FES, which some researchers associate with increased single support phase [29]. Possible explanations include a change in the balance control strategy due to restructuring sensorimotor feedback in the first hour after stimulation [30], as well as load redistribution to the “more reliable” contralateral limb in response to the first experience of a new, unusual intervention [31]. Another possible explanation is that short-term stimulation of afferent pathways can evoke transient unstable patterns, which stabilize during a course treatment [32]. Importantly, other spatiotemporal parameters (including gait speed, rhythm and double support phase) did not change significantly, which may be due to a limited permanent effect on gait kinematics while walking at a standard, “comfortable” speed.

The most likely cause of the observed changes was that the patients got tired during the FES session [33,34,35]. A 30 min multichannel functional electrical stimulation (50 Hz, 200 µs) of the large muscle groups in the thigh and lower leg creates a significant metabolic load on the paretic muscles with their disrupted motor unit recruitment [36]. Under electrical stimulation, muscle recruitment is characterized by early involvement of fast-twitch muscle fibers, which accelerates the development of peripheral fatigue and reduces voluntary muscle activity immediately after a FES session [37]. In our patient population, this phenomenon was manifested by decreased surface EMG amplitudes of gastrocnemius and hamstring on the paretic side, as well as a shortened single support phase of the paretic limb, which can be interpreted as a sign of adaptation for maintaining stability under local fatigue conditions [34]. A simultaneous increase in the movement amplitude of the nonparetic knee and a decrease in the tibialis anterior EMG on the nonparetic side fit into the model of effort redistribution for maintaining the gait rhythm at a constant speed: the intact limb takes on an additional role in stabilizing and maintaining the walking pace, while the paretic limb demonstrates economization of activity by decreasing the surface EMG amplitude under fatigue [38,39]. The contribution of the central nervous system in stroke survivors includes a decrease in corticospinal activity and worsening of muscle coordination, which can also lead to changes in muscle activity without a sustained improvement over one session [40]. Collectively, this explains why acute neuromotor changes were observed without any marked improvements in global spatiotemporal parameters [17,21]. In practice, this highlights the need to dose FES taking account of fatigue and to monitor signs of fatigue [41]. Thus, this study partially confirmed the third hypothesis: a decrease in muscle activity as a result of FES, without a phase shift. However, only two of the four stimulated muscles fulfilled this hypothesis. The other two showed no significant changes.

The absence of changes in the phase parameters of peak activities of most muscles indicates that a single FES session is not enough for a quick change in gait pattern. In the literature, the phase readjustment is demonstrated after a series of training sessions with synchronized stimulation [42], which suggests the importance of effect accumulation that enhances the plasticity of the central nervous system [29].

The study limitations include a small size of patient population, patient variability in terms of post stroke period duration and the absence of a control group.

In terms of practical significance, the study has shown that a single multichannel FES in the presented configuration is safe and causes acute neuromotor changes manifested mostly by redistribution of muscle activity and a compensatory increase in movement amplitudes in the nonparetic limb without impairment in global gait parameters. To achieve clinically significant improvements in spatiotemporal gait parameters and movement amplitudes in the paretic limb, one will probably need a course treatment with a progression of parameters and tasks focused on phase-specific training [42,43]. As an important addition to a study of persistent effects of FES, one can consider the use of bilateral stimulation synchronized with gait phases and involving both paretic and healthy limbs for even load distribution [44,45].

Functional electrical stimulation (FES) emerged approximately 60 years ago. FES is currently used to restore gait impaired by various diseases, with stroke being one of the most common of them. However, most FES application parameters remain empirical and lack a rigorous scientific basis [46]. Furthermore, the FES method remains quite labor-intensive. In our clinic, it is technically feasible to conduct only short courses of FES for patients with subacute post-stroke hemiparesis. Our previous study [25] demonstrated very limited effectiveness of 10 FES sessions conducted over two weeks. This study may provide a limited answer to the cause of this result.

Further studies should have a randomized design with a control group and a larger sample size. It would be good to test adaptive synchronization algorithms and to vary FES pulse frequency and width taking fatigue into account. This would allow assessing the extent to which the observed acute changes are transformed into sustained improvements in gait and functional independence. An additional line of research can deal with bilateral stimulation synchronized with gait phases and involving both paretic and nonparetic limbs.

The study limitations included a small sample size, patient variability in terms of duration of post-stroke period, and the lack of a detailed strategy for FES dose adjustment. In addition, limitations include the lack of a control group, observation of only one stimulation session, and the impossibility of subsequent long-term follow-up of patients. Therefore, the estimates of the effect sizes presented in this study should be interpreted with caution and considered primarily as preliminary data on the direction and magnitude of acute neuromotor changes.

## 5. Conclusions

Immediately after the single 30 min FES session, we observed a statistically significant decrease in the single support phase of the paretic limb, an increase in the amplitude of knee movements in the nonparetic limb, and a decrease in the amplitude of surface EMG of tibialis anterior on the nonparetic side and of hamstring and gastrocnemius on the paretic side. At the same time, the global spatiotemporal gait parameters and the phases of muscle activity peaks showed no significant changes.

Based on the obtained results, the direct biomechanical changes in the gait function can be interpreted as evidence of the onset of fatigue. Under fatigue, the healthy leg begins to function more actively to compensate for the muscle weakness of the paretic leg. This is probably the only direct effect of FES found in this study. The paretic limb showed no significant change in muscle activity as a result of FES.

Further studies should have a randomized design with a control group; include more subjects, optimization of FES pulse parameters taking account of fatigue.

## 6. Patents

Part of the results presented in this work may be included in a patent application related to the “Neuro Stim 2024” project. No patents have been granted or are pending at the time of publication.

## Figures and Tables

**Table 1:** Spatiotemporal gait parameters of nonparetic and paretic lower limbs before and after intervention.

Parameter	BEFORE		AFTER	
	Nonparetic	Paretic	Nonparetic	Paretic
**Gait cycle (s)**	1.55 [1.4;2.35]	1.55 [1.4;2.3]	1.55 [1.35;2.25]	1.55 [1.3;2.2]
**Stance phase (%)**	70.9 [67.6;82.6]	65.8 [62.9;70.7]	71.2 [68.2;82.4]	64.5 [63.1;70.7]
**Single support (%)**	35.2 [30.8;36.9]	30.1 [19.0;32.5]	34.4 [30.8;36.1]	28.1 [17.8;31.4] *p* = 0.0156
**Double support (%)**	35.7 [31.1;50.0]	35.9 [31.3;49.6]	35.9 [32.8;51.0]	36.3 [32.6;51.4]
**Beginning of the terminal double limb stance phase (%)**	55.2 [53.5;63.0]	45.0 [38.6;47.2]	54.7 [53.2;62.0]	44.3 [39.0;46.3]
**Foot clearance (cm)**	12.5 [11.0;13.5]	8.5 [6.5;14.0]	12.5 [10.5;13.5]	8.0 [6.0;13.5]
**Circumduction (cm)**	3.0 [2.5;3.5]	8.5 [3.0;9.5]	3.0 [2.5;3.5]	7.5 [4.0;10.5]
**Rhythm index**	0.83 [0.7;0.9]		0.81 [0.6;0.8]	
**Walking speed (km/h)**	2.0 [0.9; 2.7]		2.2 [0.9; 2.8]	

*p*-significant difference compared to the same parameter BEFORE.

**Table 2:** Amplitudes of movements in joints and myography data of nonparetic and paretic limbs before and after intervention.

Amplitude	BEFORE		AFTER	
	Nonparetic	Paretic	Nonparetic	Paretic
**Hip** (degree)	30.0 [28.0;37.0]	26.0 [19.5;32.0]	30.5 [28.5;38.0]	26.5 [17.5;35.0]
**Knee** (degree)	48.5 [43.5;56.0]	35.5 [22.0;55.0]	53.5 [46.0;57.5] *p* = 0.0234	39.0 [23.5;57.0]
**Ankle** (degree)	22.0 [18.5;27.5]	21.0 [16.0;29.0]	23.0 [19.0;28.0]	21.0 [14.0;31.0]
**Quadriceps femoris** (µV)	61.5 [51.5;101.5]	47.5 [32.0;66.5]	61.0 [49.0;82.0]	54.5 [35.5;66.5]
**Hamstring** (µV)	78.5 [68.5;155.0]	74.0 [48.0;165.5]	77.0 [57.5;148.5]	59.0 [26.0;106.0] *p* = 0.0156
**Gastrocnemius** (µV)	108.0 [81.0;129.5]	64.0 [28.0;97.5]	116.5 [85.5;143.5]	50.0 [29.0;83.5] *p* = 0.0391
**Tibialis anterior** (µV)	195.5 [122.0;237.0]	84.5 [67.0;118.5]	172.0 [98.5;193.0] *p* = 0.0078	73.0 [56.0;112.5]

*p*-significant difference compared to the same parameter BEFORE.

**Table 3:** Phases of maximum electrical activity of muscles in nonparetic and paretic limbs before and after intervention.

Phase of EMG Envelope Maximum (% of GC)	BEFORE		AFTER	
	Nonparetic	Paretic	Nonparetic	Paretic
Quadriceps femoris	13.2 [9.7;52.7]	10.0 [7.5;15.0]	11.7 [9.2;45.0]	11.5 [9.5;16.7]
Hamstring	51.2 [28.0;97.2]	15.0 [10.7;42.5]	51.7 [22.0;78.7]	13.5 [10.0;52.2]
Gastrocnemius	44.0 [22.5;52.5]	26.2 [20.2;36.2]	42.5 [40.7;47.7]	34.5 [21.2;37.5]
Tibialis anterior	10.7 [7.5;58.7]	70.2 [62.7;86.0]	8.5 [7.5;59.2]	70.5 [65.7;79.5]

## Data Availability

The original contributions presented in this study are included in the article. Further inquiries can be directed to the corresponding author.

## Abbreviations

FES	Functional Electrical Stimulation
EMG	Electromyography
CNS	Central Nervous System
TA	Tibialis Anterior
GM	Gastrocnemius
QF	Quadriceps Femoris
HM	Hamstring
IMU	Inertial Measurement Unit
MRC	Medical Research Council
EEG	Electroencephalogram
